# Bluetongue and Epizootic Hemorrhagic Disease in the United States of America at the Wildlife–Livestock Interface

**DOI:** 10.3390/pathogens10080915

**Published:** 2021-07-21

**Authors:** Nelda A. Rivera, Csaba Varga, Mark G. Ruder, Sheena J. Dorak, Alfred L. Roca, Jan E. Novakofski, Nohra E. Mateus-Pinilla

**Affiliations:** 1 Illinois Natural History Survey-Prairie Research Institute, University of Illinois Urbana-Champaign, 1816 S. Oak Street, Champaign, IL 61820, USA; sjdorak@illinois.edu (S.J.D.); jnova@illinois.edu (J.E.N.); 2 Department of Pathobiology, University of Illinois Urbana-Champaign, 2001 S Lincoln Ave, Urbana, IL 61802, USA; cvarga@illinois.edu; 3 Southeastern Cooperative Wildlife Disease Study, Department of Population Health, College of Veterinary Medicine, University of Georgia, Athens, GA 30602, USA; mgruder@uga.edu; 4 Department of Animal Sciences, University of Illinois Urbana-Champaign, 1207 West Gregory Drive, Urbana, IL 61801, USA; roca@illinois.edu; 5 Department of Animal Sciences, University of Illinois Urbana-Champaign, 1503 S. Maryland Drive, Urbana, IL 61801, USA

**Keywords:** BTV, *Culicoides* midges, EHDV, hemorrhagic disease, epidemiology, orbiviruses, reassortment, serotypes, surveillance, vector, white-tailed deer

## Abstract

Bluetongue (BT) and epizootic hemorrhagic disease (EHD) cases have increased worldwide, causing significant economic loss to ruminant livestock production and detrimental effects to susceptible wildlife populations. In recent decades, hemorrhagic disease cases have been reported over expanding geographic areas in the United States. Effective BT and EHD prevention and control strategies for livestock and monitoring of these diseases in wildlife populations depend on an accurate understanding of the distribution of BT and EHD viruses in domestic and wild ruminants and their vectors, the *Culicoides* biting midges that transmit them. However, national maps showing the distribution of BT and EHD viruses and the presence of *Culicoides* vectors are incomplete or not available at all. Thus, efforts to accurately describe the potential risk of these viruses on ruminant populations are obstructed by the lack of systematic and routine surveillance of their hosts and vectors. In this review, we: (1) outline animal health impacts of BT and EHD in the USA; (2) describe current knowledge of the distribution and abundance of BT and EHD and their vectors in the USA; and (3) highlight the importance of disease (BT and EHD) and vector surveillance for ruminant populations.

## 1. Introduction

Bluetongue disease (BT) and epizootic hemorrhagic disease (EHD) are vector-borne viral diseases caused by closely related orbiviruses (Family *Reoviridae*) that affect domestic and wild ruminants and are transmitted by insect vectors of the genus *Culicoides* [1,2]. *Culicoides* biting midges are hematophagous flies that inflict painful bites on animals and humans. Although *Culicoides* can be significant pests of humans, no pathogens are known to be transmitted to humans by *Culicoides* midges in the USA. However, multiple vector-borne diseases that affect mammal species and birds occur in the USA [3,4].

Because ruminant livestock is notably affected by BTV, while EHDV primarily impacts ruminant wildlife—in particular white-tailed deer (*Odocoileus virginianus*)—, BTV has been known longer than EHDV [5]. Bluetongue disease was first recognized in the 17th century when European sheep were introduced in South Africa [6]. However, recent phylodynamic models suggest that BTV has circulated among ruminant populations for more than 1000 years [7].

**Figure 1:**
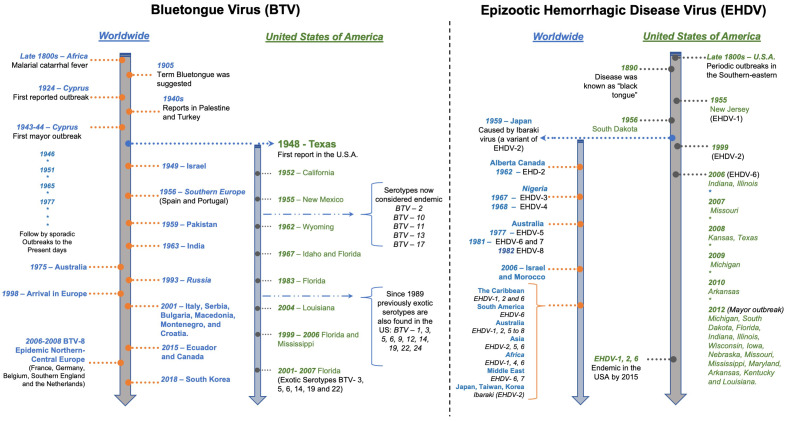
Timeline—Chronological identification of epizootic hemorrhagic disease (EHD) and bluetongue (BT) in the USA and worldwide. Data from Maclachlan et al. [5], Savini et al. [9], Mullen and Durden [18], Mertens et al. [19], Ruder et al. [20], Ruder et al. [21], Acevedo et al. [22], Tabachnick. [23], OIE-bluetongue [24], OIE-epizootic hemorrhagic disease [25], George et al. [26], and Koltsov et al. [27].

While the first BT case reports were described from sheep and cattle in South Africa, periodic outbreaks of a hemorrhagic disease affecting wild white-tailed deer in the USA have been described in the literature since the late 1800s (Figure 1) [8]. At that time, the condition was described by hunters as black tongue, which was later recognized as EHD [9]. Although not designated as BT or EHD until the mid-1900s in the USA, the description of a hemorrhagic disease syndrome affecting wild ruminants was recognized. Because overlapping clinical signs and lesions made it challenging to distinguish between EHDV and BTV affecting wildlife in the USA, the syndrome caused by BT and EHD viruses is referred to collectively as hemorrhagic disease (HD) [10].

Even though the first confirmed cases of BT and EHD were in different continents—BT in Africa and EHD in the USA—the timeframe for their confirmation was similar. Both diseases were first confirmed by the end of the 19th century [8]. By 1902, James Spreull had provided a description of bluetongue disease (also known at the time as Malarial Catarrhal Fever) affecting sheep in South Africa [11], and by 1905, Arnold Theiler demonstrated BT’s viral etiology [12]. By the end of the 1940s, epizootics of BT in sheep in western Texas were reported [13], and in 1952, the first isolation of BTV (serotype 10) was reported from sheep in California (Figure 1) [14,15]. By 1955, the viral etiology of EHD was first shown and the first EHDV serotype was identified and categorized as EHDV-1 (New Jersey strain) [16,17].

Both BTV and EHDV are closely related and genetically diverse, with multiple serotypes and distinct strains [28,29,30,31]. Epidemiological differences between these two related, but different viruses, are due to the geographic distribution of the virus serotypes and differences in the distribution of ruminant hosts and *Culicoides* spp. Worldwide, there are currently seven EHDV serotypes (EHDV-1,2, and 4 to 8) and 27 BTV serotypes (BTV-1 to 27) [32,33,34], although the number of BTV serotypes may increase as putative new BTV serotypes and strains have been described (e.g., BTV-36-CH2019 and a vaccine-derived putative BTV-28 strain) [35,36]. While vector/host interactions are impacted by climate and habitat suitability, our understanding of the proportion of wild ruminants affected by BTV or EHDV in the USA is also limited to mostly syndromic based reporting of hemorrhagic disease rather than virus isolation in a targeted population [37]. Similar knowledge gaps exist in the USA regarding the insect vectors’ geographic distribution and which *Culicoides* spp. are competent vectors; thus, the variation in vector susceptibility to serotypes and variations in their *Orbivirus* vector capacity is not well investigated. As such, there are remaining unknowns related to our understanding of the virus, vector, and host dynamics for BT and EHD [5,21,33,38].

Both BT and EHD are listed in the USA’s National List of Reportable Animal Diseases and the OIE list of notifiable terrestrial and aquatic animal diseases [39,40]. They have a negative impact not only on the local economies of regions and countries but also on the ecology and health of threatened or endangered wild ruminant populations [41,42,43]. The potential to identify the viruses responsible for EHD/BT outbreaks depends on understanding when and where ruminants are at risk of exposure to the different *Culicoides* midges that participate in the transmission of their viruses [44]. The overall goal of this review is to summarize current knowledge and potential gaps that may help improve and create an integrated surveillance system of BT and EHD in the USA. Therefore, in this review, we (1) outline animal health impacts of EHD and BT in the USA; (2) describe current knowledge of the distribution of BT and EHD and their vectors in the USA; and (3) highlight the importance of vector and BT and EHD surveillance for ruminant populations.

## 2. Animal Health Impact of BT and EHD

### 2.1. Etiology

Epizootic hemorrhagic disease virus (EHDV) and bluetongue virus (BTV) are responsible for EHD and BT, respectively. Both EHDV and BTV are members of the genus *Orbivirus*, family Reoviridae that affect domestic and wild ruminant species around the world [45,46,47]. They are linear double-stranded RNA (dsRNA) viruses with characteristic non-enveloped, icosahedral virion morphology, and structure [15,19]. While not all orbiviruses cause severe disease in animals, BTV and EHDV are the most responsible for outbreaks of economic and ecological impacts on domestic and wild ruminants. As a result, EHDV and BTV have been extensively investigated for their importance as animal pathogens, providing valuable insights for other members of the *Orbivirus* genus [19].

### 2.2. Pathology

**Figure 2:**
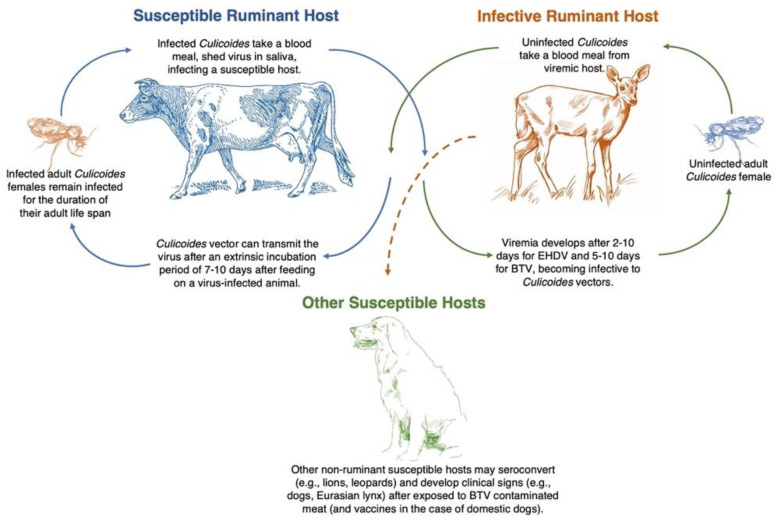
Life cycle of epizootic hemorrhagic disease and bluetongue. Data from MaClachlan et al. [17] and Ruder et al. [21]; Illustrations: Cow, Deer, Golden retriever and female *Culicoides* credit to Kerry L. Helms, Scientific Illustrator.

Animals infected with BT and/or EHD viruses show a wide range of clinical signs, from subclinical to severe clinical disease with high morbidity and mortality, especially in naïve populations [48]. Multiple factors may contribute to the severity of illness and pathogenic infections. These factors include ruminant species/breeds, age, and immune status of the animals as well as the presence of competent *Culicoides* vectors in the area, virus serotypes co-circulating, and co-infecting mammal and vector hosts (Figure 2) [48,49]. However, there is observed variation in susceptibility to infection and clinical signs between ruminant species and individual animals, and associated with different virus serogroups [50]. Thus, to better understand the transmission cycle of BTV and EHDV, there is a need to evaluate the susceptible host species’ physiology, immunology, and genetics and understand the ecology of insect vectors [18,49].

Both BT and EHD can cause clinical disease in North American ruminant wildlife, impacting wildlife conservation efforts. The clinical condition has been documented in white-tailed deer, black-tailed deer (*Odocoileus hemionus columbianus*), elk (*Cervus canadensis*), mule deer (*Odocoileus hemionus*), bighorn sheep (*Ovis canadensis*), pronghorn (*Antilocapra americana*), and mountain goat (*Oreamnos americanus*) [9,10]. Other wild and domestic ruminant hosts that have been documented to naturally or experimentally be infected with BTV and/or EHDV are presented in Table 1.

#### 2.2.1. Bluetongue Disease (BT)

The outcome of BTV infection varies both within and between ruminant host species. Some animals develop subclinical BTV infections but may still serve as a source of virus for *Culicoides* and therefore contribute to transmission (Figure 2) [5]. However, some hosts will develop clinical signs ranging from mild to moderate, with some severe cases that lead to death [10]. For example, in highly susceptible ruminant hosts like certain sheep breeds, viremia may develop after 4–6 days post infection. Animals with acute infections may die within 7–9 days, while others may survive and develop prolonged disease lasting 3–5 weeks [64]. The mortality in susceptible sheep differs between outbreaks, but could be as high as 70% [65]. Other domestic animals such as cattle and some sheep have prolonged BTV viremia lasting up to 63 days and 54 days, respectively [66,67,68]. However, this prolonged viremia rarely exceeds 60 days, and shorter viremia is usually seen [17]. For instance, experimental infections of wild ruminant hosts have reported shorter duration viremia that last up to 24 days in American bison, up to17 days for white-tailed deer, and up to ten days for elk and black-tailed deer [20,52,53,60]. A notable exception in the duration of viremia and persistence of infection for up to 111–222 days was reported in goat infected with Toggenberg *orbivirus* (BTV-25), which was first recognized among goats in Switzerland in 2008 [17,69], and in cattle transferred to Russia from Germany (BTV-14) for up to 111–138 days [27].

Clinical signs of BT disease, which are highly variable, result from erosion and ulceration in the muzzle and mouth, and edema, hyperemia, vascular congestion, hemorrhage, and tissue infarction caused by virus-mediated vascular injuries [70]. Furthermore, erosion and ulceration of the nasal and oral cavity, esophagus and forestomach, and inflammation and fluid accumulation in lungs and internal hemorrhages (cause cyanosis and the characteristic darkish blue appearance of the tongue hence the name of bluetongue) will lead to death. Clinical signs may include lethargy, altered mentation, respiratory difficulty, hypersalivation, and inappetence [10]. Some animals may show a hanging head with an arched back and tender hooves due to coronitis and laminitis that cause difficulty standing [24]. Characteristic changes have been reported for two of the most affected ruminant species. In sheep, widespread edema, especially in the lungs, is characteristic of fulminant bluetongue, whereas “extensive vascular injury results in disseminated intravascular coagulation and widespread hemorrhage” in white-tailed deer [17].

Horizontal (e.g., direct contact) and vertical (e.g., transplacental) transmission of some BTV serotypes can occur without the involvement of *Culicoides* midges [33,71]. Stillbirth and abortion have been reported in association with BTV-8 in sheep and cattle, mainly if the disease is acquired during pregnancy at the early stages of fetus development [65,72]. On the other hand, animals infected with BTV-8 during the last part of the gestation period may give birth to underweight calves RT-PCR positive to BTV-8 [71]; some animals are born with congenital deformities [24,73,74]. Fetuses/calves BTV-8 positive (either by RT-PCR or cELISA) showed lesions and clinical signs, suggesting cerebral damage (e.g., ataxia, blindness, inactivity, behavioral abnormalities (dummy calf syndrome), and hydranencephaly) [74]. Leudke and Walton (1981) reported similar results after natural breeding exposure of cattle to a BTV-11 positive bull, resulting in all calves viremic at birth and exhibiting different degrees of congenital anomalies [73]. Oral infection of neonate calves and kids after uptake of colostrum from BTV-8 infected cattle and BTV-25 infected goats, respectively, have been reported [71,75,76]. Contact transmission has been reported in experimental BTV-3 infection in white-tailed deer [77] and via artificial insemination of BTV-8 contaminated frozen semen from naturally infected bulls [50]. In addition, the spread between animals of BTV-25, 26, and 27 via direct contact with ocular or nasal secretions have also been reported [5,33].

Although not the purpose of this review, we note that other non-ruminant species have shown susceptibility to BTV during pregnancy (Figure 2). For example, transplacental infection and abortion associated with live-attenuated BTV vaccines have been reported in domestic dogs [78,79]. In addition, oral infection of carnivores by ingestion of contaminated meat has been described [6,80]. Naturally susceptible carnivore species include Florida panthers and black bears [6,81], cheetah, lions, and Eurasian lynx, among many others [40]. Reports of the development of clinical disease and death in Eurasian lynx and dogs and abortion in dogs may suggest the potentially severe consequences of BTV/EHDV for other endangered wild carnivores [6]. For some of these carnivore populations (especially threatened and endangered), the role of EHD/BT viruses and other infectious diseases that could act as additional stressors deserves further investigation, as they may impact the reproduction, survival, and movement patterns of the hosts [82]. Only applied and basic research will inform the potential role of non-ruminant species in the epidemiology of BT and EHD in domestic and wild ruminants.

#### 2.2.2. Epizootic Hemorrhagic Disease (EHD)

The outcome of EHDV infection in ruminants may differ by host species, ranging from subclinical infection to per-acute death, with some cases displaying chronic sequela after clearing the infection [9]. White-tailed deer is especially susceptible and the most affected species by EHDV, with high morbidity and mortality rates. Although other ruminant species such as pronghorn, mule-deer, and black-tailed deer (Table 1) may develop clinical signs, most EHDV infections of ruminants are mild or subclinical. Hence much lower mortality rates have been reported [9,17]. Ibaraki virus, which is a pathogenic variant of EHDV-2, is notably an exception as infection in cattle produced major outbreaks in Japan, Taiwan, and Korea (Figure 1). Other EHDV strains reported pathogenic to cattle include the Israeli strain of EHDV-7 and the 318 strain of EHDV-6 that affected dairy and beef cattle in Morocco, Tunisia, Algeria, and Jordan [9].

Studies in white-tailed deer and cattle have shown the onset of detectable viremia 3–5 days and 5–24 days post-inoculation, respectively [20,83]. Prolonged viremia of 50–60 days has been reported, with EHDV infected white-tailed deer with detectable viremia for up to 59-days and cattle for up to 50-days post-infection [2,25,84]. Experimental infection in white-tailed deer with BT and EHD viruses demonstrated the activation of the fibrinolytic and coagulation systems, resulting in disseminated intravascular coagulation [10]. The close relation between the EHD virus and the red blood cells in the late viremia can facilitate prolonged viremia in the ruminant host and contribute to the infection of the hematophagous insect vector [17,25].

In the case of EHDV infected white-tailed deer, animals may not display apparent signs of infection, and viremic animals with acute infection may die in four to 10 days [9,20]. However, sudden death has been reported. In acute EHD cases, infected animals may suffer from a rapid onset of fever and disorientation, followed by excessive salivation, lack of appetite, and weakness [19]. Similar to BT, EHD clinical signs may include a hanging head with edema of the head and neck. Animals that develop pulmonary edema or pleural effusion may exhibit labored breathing [19]. Other clinical signs include hyperemia of the mucous membranes of the conjunctiva and oral cavity as well as stomatitis accompanied by excessive salivation [83]. Painful hooves are due to inflammation of the coronary bands of the feet (coronitis) [17,25]. Ibaraki disease in cattle has been characterized by ulcerative stomatitis and dysphagia, with clinical signs showing extensive necrosis of the musculature of the upper gastrointestinal tract [17]. Abortion, reduced milk production, and infertility are other noticeable clinical signs reported in cattle [9]. Animals that develop mild infection may recover, although sequels of the tissue injury acquired during the acute phase such as damage in the oral mucosa and hoof walls may result in loss of weight, poor nutrition, and lameness [8].

As in BTV, some studies have shown evidence of EHDV oral transmission and contact transmission in the absence of arthropod vectors [85,86]. Direct contact transmission could be more important for the natural transmission of EHDV in captive populations, where high-density herds increase contact rates between hosts. Free-ranging ruminant populations may also be impacted by contact transmission [82]. Baiting and feeding of wildlife may contribute to the congregation of animals in small areas, increasing the probabilities of direct contact transmission between infected and susceptible animals [87]. Additionally, the congregation of *orbivirus* reservoir hosts may facilitate increased exposure to infected *Culicoides* midges [41]. Moreover, recognizing and understanding the role of different factors in the natural transmission cycle of HD will aid in the development of management practices and conservation policies.

### 2.3. Biological Vector: Culicoides Midges

Biting midge—tiny flies with blood-sucking habits—belong to the family *Ceratopogonidae* (Order Diptera), of which the most common are *Culicoides* spp. In North America, there are over 600 species of biting midges in 36 genera; approximately 150 species are *Culicoides* [18,88,89]. Only females of four genera—*Culicoides*, *Leptoconops*, *Austroconops,* and *Forcipomyia* (subgenus *Lasiohelea*)—require a blood meal for the maturation of the ovaries and egg production and are known to attack vertebrates [18,89].

**Figure 3:**
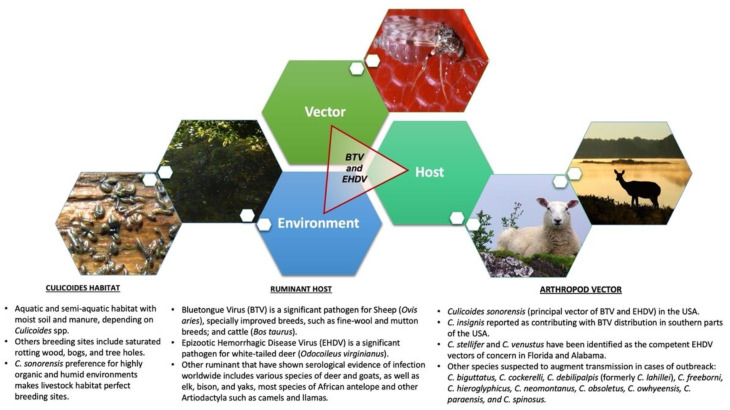
Bluetongue and epizootic hemorrhagic disease triad. Data from Ruder et al. [20], OIE-bluetongue [24], OIE-epizootic hemorrhagic disease [25], Mayo et al. [33], Ruder et al. [83], Hill and McDonald [89], McGregor et al. [92], MacLachlan and Guthrie [93], and Werner et al. [94]. Images: Sheep and thistles on Lismore, Scotland by Philippe Giabbanelli; Deer silhouette by Gary Bending; Biting midges, *C. sonorensis* by USDA; Midge larvae https://extension.okstate.edu/fact-sheets/midges-mosquitoes-and-ponds.html credit: miniturnanglers.com; Mosquitoes flying: https://pixabay.com/photos/mosquitoes-flying-insect-406491/ (accessed on 20 July 2021).

*Culicoides* midges are the only known arthropod vector for both BTV and EHDV, contributing to the life cycle and viral dissemination from infected to susceptible vertebrate hosts (Figure 3) [90]. However, only *Culicoides (Monoculicoides) sonorensis* and *Culicoides (Hoffmania) insignis* are proven vectors in the USA [88]. The incubation period of BTV in *Culicoides* lasts between 7–14 days after infection, from which *Culicoides* females become permanently infected and may transmit the virus to susceptible ruminant hosts [91].

The larval development sites of biting midges includes aquatic and semiaquatic habitats, depending on the vector species. Some *Culicoides* spp. larvae are truly aquatic (develop in both streams and ponds), while others may prefer marshes or other semi-aquatic sites with mud or moist soil and manure [18,94]. Others prefer breeding sites that may include saturated rotting wood, bogs, and tree holes [89,95]. In the particular case of *C. sonorensis*—the primary vector for BTV and EHDV in the USA—female preference for livestock habitat has been reported. The predilection of *C. sonorensis* for a highly organic and moist environment for laying eggs and larval development contributes to the transmission cycle between competent insect vector and susceptible animal hosts [23].

BTV and EHDV are found worldwide in temperate and tropical regions, depending on the arthropod vector’s geographical occurrence and distribution and susceptible vertebrate hosts. Nonetheless, the vector competence can vary for different BTV and EHDV strains within a *Culicoides* species, impacting the geographic distribution of EHDV and BTV serotypes. The lack of uniformity in the distribution of strains and serotypes may be related to the geographic distribution of competent vectors involved in virus transmission [96].

Biting midges are commonly known as *no-see-ums* in the USA, and *moose flies* in Alaska and Canada. Other common names for biting midges specific to different USA regions include *pinyon gnats* and *punkies* in the southwest and the northeastern United States, respectively. Along the Gulf Coast of Florida and Alabama, these tenacious blood-feeders of animals and humans are biting pests, also known as five-O’s, as in these areas, they are more active around 5 PM [18,89].

### 2.4. Structural and Molecular Biology

The genome of BTV and EHDV consists of 10 linear segments of dsRNA [97,98], each segment codes for seven structural proteins (VP1-VP7) and five non-structural proteins (NS1, NS2, NS3, NS3A, and NS4) [10,99]. Non-structural proteins play a role in viral replication and host–virus interactions. For example, NS4 in BTV-8 plays a role in counteracting antiviral responses of the host [99]. The three major layers of protein are the sub-core (VP3), the core-surface layer (VP7), and the outer capsid (VP2 and VP5) [19]. Enclosed in the inner core are three minor proteins (VP1, VP4, and VP6) and the dsRNA genome [10]. Both serogroup- and serotype-specific antibodies develop according to the proteins in the different layers of the virus [10]. While VP7 (the major protein on the core’s surface) and VP3 (the major sub-core protein) have been associated with serogroup-specific antibody specificity, serotype-specific antibodies are primarily directed to determinants on VP2 [10]. Furthermore, cell attachment and entry mechanisms are associated with VP2 mediate infection in mammalian but not in *C. sonorensis* cell lines [96].

To date, seven serotypes for EHDV and 27 serotypes for BTV have been confirmed [32,96,100,101,102] including two genetically distinct BTVs identified from healthy goats in Italy (X ITL2015) [103] and goats and sheep in China (XJ1407) [104]. There are genetically different strains of the virus throughout the world, and the field strains may differ on phenotypic properties like virulence and transmission potential [65]. Mutation (a change in DNA sequence) and reassortment (the genetic recombination between different virus serotypes/strains co-infecting a host cell) of RNA viruses contribute to the genetic diversity among field strains, changing transmissibility, pathogenicity, and causing altered virulence in susceptible ruminant hosts [33,105].

Moreover, the diversity of EHDV/BTV is associated with the reassortment of segments of the viral gene during the co-infections (with more than one virus strain) in either ruminant or vector host cells that lead to genetic change [65]. Furthermore, the appearance of antigenically new viruses or genetic variants through mutations in either insect or animal hosts can develop due to genetic drift (mutation of individual genes) during virus replication [5,17]. Worldwide, the evolution of different field strains is driven by genetic drift, genetic reassortment between viruses within each genus or serogroup, intragenic recombination, and the selective evolutionary pressure to establish genetically distinct virus strains in diverse epidemiological systems [17,65].

Biological and genetic properties of the newly identified BTV serotypes (e.g., BTV-25, 26, and 27) revealed changes in virulence and pathogenicity compared to the 24 historical BTV serotypes (BTV-1 to 24) [5]. Besides, the mode of propagation differed not only between the novel (BTV-25, 26, 27) and traditional (BTV-1 to 24) serotypes, but also among the novel serotypes. Some of the experimental studies may explain the mechanism of transmission for some strains. For example, experimental studies with BTV-25 have been complex, as the traditional methods used for in vitro propagation of BTV 1–24 (e.g., cell culture systems or embryonated chicken eggs (ECE)) are not effective [106], suggesting difficulties for the virus to propagate on vertebrate hosts. BTV-26 and BTV-27, on the other hand, can be propagated in vitro although with limited growth capability—BTV-26 can propagate in mammalian cell lines but not in *Culicoides*-derived cells (Kc) [5]. The genome segments Seg-1/VP1, Seg-2/VP2, Seg-3/VP3, and Seg-7/VP7 of the BTV-26 restrict the ability of the virus to infect and replicate in *C. sonorensis* cell lines (Kc) [96]. Furthermore, BTV-27 does not grow in ECE or cell cultures (e.g., Kc, African green monkey kidney (Vero) cells, and baby hamster kidney (BHK) cells) [5]. These studies raise more questions about viral adaptation to hosts and competent vectors, and their capacity to adapt and become established in different ecosystems.

Phylodynamic and phylogeographic models have provided fundamental insights into arbovirus spread across the globe, yet for many arboviruses, these models have yet to be utilized [7]. It is not until recently that phylodynamic models employed to quantify the evolutionary characteristics, spatiotemporal origins, and multi-host transmission dynamics of BT globally estimated that BTV has circulated among ruminant populations for at least 1000 years (but probably more than 2000 years). These models also identified China and India as the ancestral countries from where BTV emerged and dispersed (and not Africa as it was believed), and goats as the ancestral host for the virus (and not sheep) [7]. Nonetheless, the ability to identify the virus and the competent vectors involved in the outbreaks as well as a sustainable mechanism for long term data, will allow for the evaluation of changes in serotype adaptation across space and time and the epidemiological models that will ultimately inform risk of severe outcomes in susceptible hosts.

### 2.5. Impact

Infectious diseases such as BT and EHD have an economic impact worldwide, as losses of animal, bans on animal movement and trade restrictions, and prevention and control expenses affect both individual farmers and the broader agricultural industry as well as management and conservation efforts directed at wildlife [22,24]. The distribution of BTV and EHDV depends on both mammalian and biological vector host geographic distribution and abundance. However, morbidity and mortality rates may depend on mammalian host susceptibility to the different serotypes circulating in each geographical area [24,25]. However, the evolutionary rates that vary between enzootic and epizootic transmission are unknown in the USA, as there is no detailed information of the circulating serotypes in different geographical areas.

For livestock, mortality rates for BT and EHD may range between 0–100% depending on the ruminant host and prior infection, affecting the regions and countries’ economies depending on the severity of the outbreak [24,25]. However, morbidity cost is related to the care for sick animals (e.g., veterinary cost and animal support) and reduced productivity of affected animals in livestock operations (e.g., weight loss, reduced milk yield, and abortion).

There is no cure or treatment for BTV and EHDV; therefore, the goal of BT and EHD management is to prevent virus spread into unaffected areas and clinical disease in ruminant hosts [4]. For example, BT has been listed as a notifiable animal disease by the Office of International Epizootics [24,25,40]. Restrictions in the trading and movement from enzootic regions of livestock and their products—including those that could be vertically transmitted, such as fetal bovine serum and fetal tissue—are suggested to avoid introducing the viruses to new places [88].

## 3. Prevalence and Distribution of BT and EHD in the USA

Historically, EHDV/BTV has occurred in the southeastern, central, and western USA. However, during the last 20 years, these viruses have spread northward within the USA territories, with cases reported across the upper Midwest and the northeastern USA (Figure 1) [38,107]. The distribution of EHD/BT depends on the occurrence of competent *Culicoides* vector species. The home range of *Culicoides* midges was historically maintained between latitude 35° S and 40° N [108], however, changes in the global range of vectors and distribution of BT and EHD have shown a northward expansion where the diseases are maintained between latitudes 35° S and 50° N [108,109].

Changes in the distribution of EHDV/BTV and the pathological effect of newly described serotypes on domestic ruminants seem to increase the importance of BT and EHD worldwide. In the USA, two EHD serotypes (EHDV-1 and -2) and five BTV serotypes (BTV-2, -10, -11, -13, -17) were considered historically endemic. Recently, since 1989, ten previously exotic BTV serotypes (BTV–1, 3, 5, 6, 9, 12, 14, 19, 22, 24) have also been found in enzootic southeastern areas of the USA (Figure 1) [5,33]. Furthermore, the once exotic EHDV-6 identified in wild white-tailed deer populations in Indiana and Illinois in 2006 is now considered endemic [110]. In addition, new strains of BTV (serotypes 1, 2, 4, 8, 9, 16), previously found only in sub-tropical regions, have appeared in Europe [43]. Thus, the changes in the distribution of EHDV/BTV suggest a worldwide phenomenon [43,59].

Increased occurrence of vector-borne diseases during the last decades have been linked to changes in environmental conditions such as an increase in temperature and rainfall [111]. For example, spatiotemporal models used to analyze 15 years of drought data collected in the eastern United States found the severity of drought and latitude (especially for areas over 33° N latitude) as strong predictors of the occurrence of EHDV/BTV outbreaks in areas with enzootic EHD events [107]. Previous studies conducted in North Dakota, South Dakota, and Nebraska found altitude and latitude as risk factors for BTV in cattle [112]. The effect of the virus serovar and the arthropod vector genotype and environmental factors such as temperature and humidity have been identified as contributing to the mechanisms that influence the competence of *Culicoides* for viral dissemination [113,114,115]. Furthermore, temperature and humidity can affect the extrinsic incubation period of the virus in *C. sonorensis* and vector competence and survival.

Wittmann et al. (2002) found that increasing temperatures reduced the extrinsic incubation period for both BTV and EHDV, which can, in turn, facilitate the transmission of orbiviruses; although, rising temperatures also reduce vector survival [59,115]. In addition, differences in vector competence between EHDV and BTV were described as higher temperatures (e.g., 27–30 °C) increase vector competence for EHDV (serotype 1) but not for BTV (serotypes 10 and 16) [115]. When temperature and humidity were evaluated together, both high humidity/temperature and low humidity/temperature were detrimental for vector longevity [115].

As with other vector-borne viruses, there is a strong link between vector-borne disease transmission and weather and climatic variables. The effect of wind velocity, rainfall, and temperature are among the climatic variables that have been long recognized to affect *Culicoides* abundance and activity [116]. The drought conditions and the record high temperatures reported in 2012 in the USA—the warmest year in the 1895–2012 period according to the National Climatic Data Center [117]—have been suggested as a contributing factor for the 2012 EHD outbreak [59]. A temporal association of regional EHDV/BTV reported cases expanding to northern latitudes had been demonstrated and correlated with the regional drought conditions [38]. Although there could be an apparent contradiction between drought and the necessity of *Culicoides* spp. for aquatic/semiaquatic habitats, lower water levels during drought create a warmer and shallower water source, and expose the fresh mud that is an ideal habitat for the reproduction of some *Culicoides* spp. [94]. Additionally, the limited water availability promotes the congregation of animals to fewer water sources, increasing the number of animals from which Culicoides can feed. Animal congregation further supports midge reproduction by creating mud disturbance enriched with fecal matter at these sites [94,118]. This is important as manure has been identified as a suitable substrate for biting midge propagation and reproduction [94].

Spatial analysis of a more recent EHDV/BTV outbreak in the eastern USA in 2017 identified associations of the outbreak with a specific physiographic region, the Appalachian Plateau [119]. The statistically significant spatial dependence of the reported EHDV/BTV cases suggested that long-term weather patterns in the eastern USA, which are controlled by geology and climate, could have contributed to the 2017 hemorrhagic disease outbreak. Thus, not only abrupt changes in environmental conditions (e.g., drought), but also spatial characteristics of a specific physiographic region, may influence hemorrhagic disease epidemiology, highlighting the importance of continuing and increasing surveillance efforts to identify and better understand the contributing factors to hemorrhagic disease outbreaks in specific areas of the USA [119].

The expansion of BTV-8 into northern Europe demonstrates a shift from the historically recognized geographic distribution area of BTV to previously disease-free areas (with the potential of the insect vector to overwinter within its new habitat) [91,116,120,121]. Furthermore, the expansion of BTV into areas where the main vector, *C. imicola*, is rare or absent suggests the involvement of previously unidentified indigenous European *Culicoides species* (e.g., the *Culicoides obsoletus* group and *Culicoides pulicaris* group) that were widespread and abundant [121]. In addition, the expansion of the geographic distribution of BTV to north-central Europe starting in 2006 has been linked to climate change and shifting wind patterns affecting *Culicoides* distribution and virus transmission [122,123].

In the USA, a shift in the occurrence of *Culicoides* midges and the persistence of viruses overwinter months also suggest changes in adaptation and the dynamics of EHDV/BTV. Distribution of EHDV/BTV outside of the normal range of the main vector, *C. sonerensis*, might be related to the occurrence of different *Culicoides* midges serving as viral vectors, as is the case of *C. insignis*, which is known to participate in the life cycle of BTV-2 in Florida [88]. A northwestward geographical range expansion of *C. insignis*—a neotropical species previously known for a northern-most range up to peninsular Florida—was confirmed during a survey conducted between 2007 and 2015, as *C. insignis* was detected in five additional USA states [88]. Furthermore, interseasonal (overwinter) maintenance of BTV was associated with prolonged survival of *C. sonorensis* midges in California [124]. The increase of the vector’s home range to northern latitudes (either by changes in wind, rainfall, or temperature), coupled with changes in climate that leads to increased temperatures and enable longer EHDV/BTV transmission seasons and interseasonal maintenance of viruses, represent a risk factor for naïve populations [107,111].

While *C. sonorensis* and *C. insignis* are confirmed competent vectors for EHDV/BTV in the USA, there is strong evidence that suggests that other species can be involved in the transmission of EHDV/BTV viruses. However, in order to elevate other *Culicoides* spp. to confirmed vectors, a set of criteria needs to be fulfilled. There are four criteria defined by the World Health Organization (WHO 1967) [125] that are used as guidelines for incriminating an insect as a vector of a disease agent or pathogen: (1) repeated recovery of the pathogen from blood free, wild-caught insects; (2) demonstration in a control environment that the insect can become infected via a blood meal from a viremic vertebrate host or an artificial substitute; (3) demonstrate the transmission of the pathogen to a susceptible vertebrate host after the bite of an infected insect vector; and (4) demonstration on the field of the contact of the insect vector and susceptible vertebrate host populations [9,20]. In North America, *C. sonorensis* has been reported as the primary vector of EHDV and BTV, as studies have demonstrated its implication as a vector in the field (via isolation of the pathogen from field-collected vectors and demonstrated contact between vector-ruminant host) and the laboratory (by the demonstration of vector infection after a blood meal from an infected host and subsequent transmission of the pathogen to a susceptible host) [9]. Moreover, *C. sonorensis* complies with the four criteria that demonstrate its status as the primary insect vector for BTV/EHDV in the USA.

Vector competence studies are essential to establish the presence of primary vectors (critical to maintenance of the viruses in the host population) and secondary vectors (candidate vectors that may contribute to virus dissemination, helping to explain changes in distribution/geographic expansion of BT and EHD). Based on the four guidelines defined by WHO, vector competence—the capability of an insect vector species to become infected after a blood meal from a viremic/infected vertebrate host—can be estimated by using the vector implication criteria 2 and 3 of the of the World Health Organization [20]. In search of other potential *Culicoides* vectors, accurate verification of ruminant host-*Culicoide* vector contact and subsequent blood meal analysis of the vector is needed. For example, *C. debilipalpis* (formerly *C. lahillei*) are considered candidate vectors for EHDV as laboratory studies have demonstrated that they can become infected after a blood meal and do come in contact with susceptible host ruminants in the field [9]. Furthermore, laboratory studies have shown that *C. venustus* can get infected with the pathogen after a blood meal, while isolates of the pathogen from blood-free field-collected *C. mohave* have also been reported [9]. Other *Culicoides* species regarded as potential vectors in Florida include *C. stellifer, C. debilipalpis, C. venustus, C. pallidicornis,* and *C. biguttatus*, as these species have been demonstrated to come into contact with susceptible ruminant species in the field [92]. However, all four criteria from the World Health Organization need to be met to elevate vector species to competent vectors of BTV and EHDV.

## 4. The Importance of Surveillance for Ruminant Populations

The prevention of EHD/BT in livestock depends on understanding when and where domestic and wild ruminants are at higher exposure risk to circulating EHD/BT viruses, which relies on the distribution of *Culicoides* vectors [126]. For instance, goats are a major host for BTV, as demonstrated by the extended periods of viremia [69,76], high virus replication, and absence of clinical disease [127] although goats are less likely to be important for cross-species transmission [7]. However, the role of sheep in the transmission to other susceptible species and the role of cattle as reservoirs for BTV remain significant, while wild ruminants have a substantially lesser role in the transmission of BT virus [7]. These results agree with recent findings in the USA, where intensive production systems played a role in maintaining EHD/BT viruses in livestock [128], which could consequently contribute to infection in wild ruminant populations.

Suggesting higher animal density as a significant contributing factor for hemorrhagic disease virus maintenance, a recent study in Florida compared EHDV antibody prevalence between a farmed herd of white-tailed deer kept in breeding pens, white-tailed deer living in the surrounding forest (captive white-tailed deer maintained in hunting pens that also housed non-native ruminants), and wild white-tailed deer adjacent to the farm. In this study, the higher seroprevalence of EHDV was found in captive herds—both in farmed white-tailed deer herds and in managed preserve herds. In this particular case, besides higher density, the presence of non-native ruminants that may act as amplifying hosts for EHDV such as elk (*Cervus canadensis*) was suggested as increasing the prevalence in these populations [128]. Interestingly, as indicated by the authors, despite the use of chemicals to control the vectors, the exposure of farmed white-tailed deer to EHDV was higher than in wild ruminants living near the area [128]. A higher relative abundance of *Culicoides* has been positively correlated with proximity to areas where host density is elevated (e.g., big game preserve, farms). Thus, a high density of animals may contribute to a higher density of vectors, suggesting increased transmission potential in these and adjacent areas [129]. While not evaluated in these studies, host density may impact habitat cover [130] and, in turn, have an impact on the phenology of competent vectors [131].

Host density is not the only factor that could be contributing to the spread of orbiviruses. Host preference patterns of biting midges at a big game preserve in Florida identified five *Culicoides* spp. that showed variations in preference and avoidance patterns for seven Bovidae and seven Cervidae hosts [92]. For instance, *C. biguttatus* and *C. pallidicornis* showed preferences for Père David’s deer (*Elaphurus davidianus*) and white-tailed deer, respectively. While *C. stellifer* showed a preference for Cervus species in general, it displayed a relative avoidance of white-tailed deer and Bovidae. *Culicoides debilipalpis* also avoided all Bovidae but preferred white-tailed deer [92]. This study highlights the potential impact of animals (both native and non-native) on *Culicoides* composition at these sites.

In Louisiana, a study that evaluated the presence of EHDV/BTV antibodies in captive populations of cattle and white-tailed deer identified higher infection rates among cattle [132]. This study maintained white-tailed deer in fenced enclosures, and the cattle herd was rotated among open pastures. Two possible explanations of the findings were that cattle might be more attractive to *Culicoides* midges than deer as they release higher carbon dioxide levels and have larger body sizes [132]. However, other factors could be contributing to the host-vector dynamic in this particular geographical region. Further evaluation of chemical cues affecting host-seeking *Culicoides* and changes in *Culicoides* habitat could help us understand the ecological dynamics that may contribute to EHDV/BTV transmission. All in all, the cattle in this study showed much higher rates of EHDV/BTV exposure than deer, which in turn suggest their role as a source of BTV/EHDV infection [132]. For instance, although BTV and EHDV seroconversion of cattle and deer were reported in Louisiana, *C. sonorensis* was not among the *Culicoides* spp. captured in this study, and none of the *Culicoides* captured were EHDV positive [132]. Previous studies have shown differences in host-seeking activity of various *Culicoides* spp. to different pasture livestock [126]. Further investigation and a better understanding of the *Culicoides* participating as competent vectors of different epizootics is needed to increase our knowledge of the epidemiology of EHDV/BTV in various areas in the USA. Integrated surveillance approaches that incorporate sentinel animal studies, serological surveys in wildlife and livestock, and disease reporting, coupled with vector surveillance, are required to evaluate the ruminants’ risk of acquiring EHD/BT viruses.

Furthermore, routine and systematic surveillance will help define epidemiological zones in the USA (for instance, areas of enzootic and epizootic transmission) and identify areas at risk for EHDV/BTV incursion. These zones can be classified based on the impact to the hosts as enzootic (zones with mostly sub-clinical cases), epizootic (clinical cases occur at regular intervals), and incursive (zones with extended periods of time between disease cases, but, when these occur, the epizootic is often extensive) [49]. Nevertheless, outdated or incomplete knowledge of the geographic distribution of the EHD/BT virus and its competent vectors—and in some cases, the complete absence of reporting cases to a centralized passive surveillance program like the National Hemorrhagic Disease Survey of the Southeastern Cooperative Wildlife Disease Study (SCWDS), College of Veterinary Medicine, University of Georgia [33], and the National Veterinary Services Laboratory (NVSL)—stimulate the need to increase efforts related to surveillance, research, management programs, and the development of communication efforts to the public to create awareness of mortality events, disease risk, and anticipated outcomes [133].

As previously demonstrated, genetic reassortment between virus strains may result in new viruses with unique biological properties. For instance, during the highly virulent BTV-8 outbreak in Europe in 2006, it was noticed that not only sheep but also goats and cattle suffered from acute disease and that BTV-8 transplacental dissemination was not associated with the use of vaccines—a pathogenic property previously associated only with the vaccination of pregnant animals [65,134,135]. Furthermore, there was a higher tendency of BTV-8 to be secreted in the semen of naturally infected bulls and rams, while indigenous *Culicoides* not previously identified as potential vectors for BTV were implicated in the 2006 BTV-8 outbreak and subsequent expansion into northern Europe [65,136,137]. These examples show the importance of determining unusual features compared to previous outbreaks to determine epidemiological changes in EHD/BT that will lead to timely appropriate intervention and management of infectious diseases (e.g., developing inactivated vaccines to reduce animal mortality and a timely change in the development of animal movement protocols).

Thus, surveillance with whole genome sequencing may be a way to better understand the genetic makeup of the viruses causing the outbreaks. In turn, it facilitates studies to evaluate the potential alteration of viral phenotypes to better understand EHD/BT viruses in the USA. Detailed knowledge of molecular determinants such as genetic markers that affect cell tropism and other viral characteristics that could contribute to the rapid dissemination of new strains (e.g., that may aid in the participation of indigenous *Culicoides* spp. not previously associated with EHDV/BTV dissemination) is essential for management of captive ruminants.

Domestic cattle have been implicated as important reservoirs for BTV and EHDV, while wild ruminants may act as important reservoirs for EHDV but only as potential reservoirs for BTV, however, the relative role of each ruminant species in EHDV/BTV circulating in the USA remains unsolved [7]. While the epidemiological process of BT and EHD is similar, a complete understanding of the interaction between host, virus, and vector—each of which may be affected by environmental variables—is needed to determine the risk of viral exposure that leads to disease outbreaks (Figure 3). Moreover, a systematic collection of data will allow for the timely implementation of measures to prevent and control BT and EHD outbreaks in livestock. These data will help to explain epidemiological trends and predict changes in risk for these diseases: (1) at a local scale by providing information on when and where ruminants are at higher risk of exposure to *Culicoides* that could be carrying EHD/BT viruses; and (2) at the state and national level by providing information necessary to identify areas where cases have occurred previously, and areas where new cases have been reported, thus the movement of livestock and relocation of wildlife can be regulated and properly managed. Combining ruminant host and *Culicoides* surveillance data might be used to predict the expansion of endemic areas affected by EHDV/BTV. Furthermore, *Culicoides* surveillance and evaluation of potential competent vectors will continue to inform what serotypes are better adapted to a vector and host, which is a valuable tool to explain and predict epidemiological trends or temporal shifts of BT and EHD.

## 5. Conclusions

Current and accurate information on when and where animals are at risk of exposure to EHD/BT viruses helps target research efforts toward understanding different determinants of serotype occurrence in several USA regions. It also offers the opportunity to evaluate virus, vector, host, and environmental condition that allows different EHDV or BTV serotypes to become established in a geographic region. In addition, advances in the pathogenesis of the viruses, immune response in the host, vector competence, impact of weather, and changes in habitats on the vectors’ survival may influence the epidemiology of EHDV and BTV. Over time, these advances may contribute to the development of mechanisms to manage disease outbreaks. The disease distribution maps have been generated based on agency reports and previous peer-reviewed literature. However, the lack of ongoing and systematic BTV and EHDV surveillance efforts and the limited understanding of which *Culicoides* serve as competent vectors makes it challenging to develop accurate distribution and risk-associated maps. A systematic sustained, and comprehensive disease-virus-vector surveillance program for EHDV and BTV in wildlife and livestock would help to fill in research gaps.

Although researchers have been describing and identifying BT and EHD in the USA for more than a century, the approaches to surveillance for captive and wild ruminants differ considerably. In addition, the conditions and potential to survey *Culicoides* near the hosts and the options to sample and determine exposure to EHD/BT viruses in the hosts also differ. Collectively all these sources of variation complicate comparisons over time and sampling areas. Moreover, the lack of uniform data collection methods across the country renders the data with limited value to generalize over different habitats as there could be under-reported cases, leading to the under-estimation of the actual distribution of EHD/BTV [59]. Despite this, the data used from published studies and from the National Hemorrhagic Disease Survey of SCWDS to develop EHD/BT distribution maps revealed significant geographic expansion of EHD/BT during recent decades. The observation emphasizes the importance of surveillance in the ruminant host at risk of infection (both captive and wild ruminants).

Furthermore, the identification of viral mutation and reassortment is essential as this is the primary mechanism for the emergence of virulent RNA viruses and the interspecies transmission that may lead to epizootic virus strains [105]. Surveillance in Europe has provided the ability to identify EHDV/BTV strains with the potential of rapid spread and overwintering capabilities and to discriminate between non-virulent and virulent field strains [65]. Thus, a better understanding of the gene pool of circulating viruses in enzootic areas in the USA and the repercussions of the introduction of live-attenuated viruses that could contribute to genetic reassortment is needed for future prediction of the emergence of BT and EHD. Therefore, there is a need for whole-genome sequencing to understand the genetic makeup of these viruses in the USA. This is critical information for policymakers and wildlife managers as it will help inform of the impact that new EHD/BT outbreaks are likely to have and will aid in conducting cost-risk analysis before the implementation of the control measures [65] mostly available for livestock ruminants. Surveillance to identify distribution of the virus in a state and within regions may allow for the identification of areas of virus introduction.

National maps showing the abundance of ruminant-seeking *Culicoides* midges provide improved data for assessing the likelihood of ruminant encounters with important *Culicoides* spp. for each ruminant host (e.g., captive vs. free-ranging); however, depending on the *Culicoides*, such maps are either not current or non-existent. Moreover, *Culicoides* surveillance can provide objective and quantifiable data that reconcile misperceptions of risk and refocus attention to underrecognized vector species of increasing importance for the transmission and expansion of BT and EHD. However, to establish the distribution of vector-competent species of *Culicoides* midges and the vectorial capacity of the populations of resident midges previously considered as not competent for hemorrhagic disease dissemination, field and laboratory experiments should be implemented to determine vector status [5].

While *Culicoides* are the only arthropod vectors of EHDV/BTV, other modes of transmission (e.g., direct contact and oral transmission) have been reported for some EHDV/BTV strains [5,7,33,71,75,85,86,132]. Moreover, despite the presence or absence of arthropod vectors, EHDV/BTV surveillance should be conducted, and systematic reporting methods should be implemented as part of the managing of livestock and wildlife populations, especially where the movement of nonindigenous species, importation of naïve animals, and/or reintroduction of animals back to their native range are being used [41,86].

All in all, national *Culicoides* and EHDV/BTV surveillance efforts can improve our understanding of geographic variation in risk factors for EHDV/BTV, and efforts to build such a program have increased in recent years. Nonetheless, the sustainability of BTV, EHDV, and *Culicoides* midge surveillance programs depends on improving their financial/fiscal support and coordination and efficiency to render them cost-effective. In addition, the final results of implementing systematic animal and vector surveillance can provide the necessary data for more extensive coordination among local, state, federal agencies and universities as well as agricultural agencies involved in assessing animal diseases and animal well-being.

## Figures and Tables

**Table 1:** Reported BTV and/or EHDV infection with or without clinical signs in wild and captive ruminant host species in the USA.

Ruminant Species			Detected (*✓* )		References
Family	Common Name	Latin Name	BTV	EHDV	
Cervidae	White-tailed deer	*Odocoileus virginianus*	*✓*	*✓*	[9,10,20,41,51]
	Mule deer	*Odocoileus hemionus*	*✓*	*✓*	[10]
	Black-tailed deer	*Odocoileus hemionus columbianus*	*✓*	*✓*	[20,52]
	Elk (wapiti)	*Cervus canadensis*	*✓*	*✓*	[10,41]
	Rocky Mountain Elk	*Cervus elaphus nelsoni*	*✓*	*✓*	[53,54,55]
	Axis deer	*Axis axis*	*✓*	*✓*	[10,51]
	Fallow deer	*Dama dama*	*✓*	*✓*	[10,41,51,56]
	Sika deer	*Cervus nippon*	*✓*	*✓*	[10]
	Yaks	*Bos grunniens*		*✓*	[57]
	Père David’s deer	*Elaphurus davidianus*		*✓*	[41]
	Moose	*Alces alces*		*✓*	[58]
Bovidae	Cattle	*Bos taurus*	*✓*	*✓*	[9,56,59]
	Mountain goat	*Oreamnos americanus*	*✓*		[9,10]
	Bison	*Bison bison*	*✓*	*✓*	[10,59,60]
	Blackbuck antelope	*Antilope cervicapra*	*✓*	*✓*	[10]
	Gerenuk	*Litocranius walleri*	*✓*		[61]
	Bighorn sheep	*Ovis canadensis*	*✓*	*✓*	[9,10,59]
	Dall sheep	*Ovis dalli*		*✓*	[58]
	Bongo antelope	*Tragelaphus eurycerus*		*✓*	[41]
	Roan antelope	*Hippotragus equinus*		*✓*	[41]
	Lesser kudu	*Tragelaphus imberbis*		*✓*	[41]
	Dama gazelle	*Nanger dama*		*✓*	[41]
Antilocapridae	Pronghorn	*Antilocapra americana*	*✓*	*✓*	[10,62]
Camelidae	Alpaca	*Vicugna pacos*	*✓*		[63]

## Data Availability

Not applicable.
